# Co-creating a social science research agenda for Long Covid

**DOI:** 10.3389/fpubh.2025.1654488

**Published:** 2025-11-06

**Authors:** Oonagh Cousins, Maaret Jokela-Pansini, Nisreen A. Alwan, Ella Barnard, Jenny Ceolta-Smith, Jo Dainow, Caroline Dalton, Gail Davies, Mark A. Faghy, Eileen Gilmour, Ian Patel, Ondine Sherwood, Lotus Westerhof, Beth Greenhough

**Affiliations:** 1 Long Covid Support, London, United Kingdom; 2 School of Geography and the Environment, University of Oxford, Oxford, United Kingdom; 3 Department of Geography, University of Zurich, Zürich, Switzerland; 4 School of Primary Care, Population Sciences and Medical Education, Faculty of Medicine, University of Southampton, Southampton, United Kingdom; 5 University Hospital Southampton NHS Foundation Trust, Southampton, United Kingdom; 6 Patient Author, London, United Kingdom; 7 College of Health Wellbeing and Life Sciences, Sheffield Hallam University, Sheffield, United Kingdom; 8 Geography, Faculty of Environment, Science and Economy, University of Exeter, Exeter, United Kingdom; 9 Human Science Research Centre, University of Derby, Derby, United Kingdom; 10 Patient Author, Kilmarnock, United Kingdom; 11 Patient Author, North Berwick, United Kingdom; 12 Long Covid SOS, London, United Kingdom; 13 Patient Author, Harderwijk, Netherlands

**Keywords:** Long Covid, COVID-19, social science, agenda-setting, survey, PPI, stakeholder engagement, co-creation

## Abstract

**Introduction:**

Our objective was to understand how social scientific research could best address the needs and concerns of patients, families, carers, healthcare professionals, academics, private and public sector professionals, and volunteers from Long Covid charities and support groups and people with lived experience of Long Covid. We worked with different stakeholders to develop a list of research priorities that particularly focused on social science as this is where our collective expertise lies, but similar methods could also be used to set research priorities in the natural sciences, medicine or the humanities.

**Methods:**

We used purposive sampling and conducted two online surveys. The first online survey (*N* = 57) asked participants to identify their top five questions of concern, which resulted in a list of 253 questions. These questions were then consolidated, refined and edited down to 55 questions, categorized by topic. In the second survey (*N* = 66), we asked participants to select and rank their top 10 questions from this refined list. The final output was a ranked list of nine questions based on those prioritized by at least 50% of the respondents.

**Results:**

Nine research questions were developed concerning (i) treatments, therapies, and strategies; (ii) financial support; (iii) repeated reinfections; (iv) training of healthcare professionals; (v) mental health impact; (vi) future of research funding; (vii) airborne transmissions of COVID-19; (viii) developing therapeutics informed by patients’ experiences; and (ix) socioeconomic impacts of Long Covid. Many of the issues raised mirror those discussed in previous work in the UK and internationally, but additional novel themes emerged, underscoring the value of this collaborative approach.

**Conclusion:**

Our survey revealed the value of including the voices of diverse individuals affected by Long Covid and those working in this area and highlighted priorities for social science in the field of Long Covid research.

## Introduction

The term “Long Covid” is used to describe post-acute and, potentially, long-term disabling health effects that follow a SARS-CoV-2 infection and are not explained by another cause. Other terms that are commonly used include postcovid19 condition, post COVID, post-acute COVID, post-acute sequelae of SARS- CoV-2 infection (PASC), chronic COVID, and long-haul COVID (1). It is estimated that about 6 to 7% of adults and roughly 1% of children with acute COVID-19 symptoms experience Long Covid, resulting in approximately 400 million people affected by the condition since the start of the pandemic worldwide (2). This is likely a conservative estimate, considering reduced testing for acute COVID-19 infection, the possibility of the condition arising from asymptomatic infections, and the potential for additional risks from reinfection or long-term latent effects that are not yet fully understood (3). Long Covid and its effect on individuals’ health can severely affect continuing daily activities, including the ability to continue domestic chores, leisure, social activities, work, selfcare, childcare, and mental health (4) and given its complexity and widespread occurrence, it also poses significant challenges to health systems and economies. Consequently, Long Covid represents one of the most considerable global health challenges of our time. Scientists have made significant progress in some research areas, but much work remains to understand the illness’s various impacts on patients’ health, its underlying biological mechanisms, and potential treatments (2).

Addressing and understanding Long Covid demands an interdisciplinary approach that goes beyond the biomedical perspective. It is well established that health and illness are shaped by social, economic, and political determinants (5), a reality that the COVID-19 pandemic has powerfully highlighted (6). Long Covid not only affects physical health but also disrupts social roles, economic participation, and mental and emotional well-being. For example, many people have reported feelings of loneliness and exclusion and shifts in identity due to the changes in their health as well as in their work- and social life (7). Building on the idea of interdisciplinarity, our paper aims to explore how social scientific research particularly may contribute to understanding certain research topics and questions on Long Covid, without assuming that these research topics are relevant for social scientific inquiry only.

Previous social scientific research has highlighted a number of key concerns with respect to addressing patients’ experiences and government responses (or lack thereof). First, Long Covid is a complex, multisystem pathophysiological condition, which can present with multiple sequelae across almost all systems and organs, including the cardiovascular, nervous, endocrine, immune, reproductive, and gastrointestinal systems. It is characterized by symptoms such as fatigue, breathlessness, cognitive dysfunction (often referred to colloquially as ‘brain fog’) and pain, and has many health effects in common with other post-acute infection syndromes (8). Some progress has been made in understanding the underlying mechanisms, which are likely numerous given the complexity of the condition, but much work remains to be done. Diagnostic and treatment options remain limited, and there is currently no evidence-based gold standard treatment (9). The care provided to individuals with Long Covid varies widely across different settings and practitioners, often failing to respond to patients’ needs (10).

Second, in addition to its effects on individuals’ health outcomes and daily lives, Long Covid represents a significant economic and public health crisis, which is evident in its considerable strain on economies and healthcare systems. At an individual level, it affects people’s capacity to work, resulting in significant financial hardship, depleted savings for those who had any, and food and housing insecurity (11). Studies show a notable reduction in work capacity and employment among those affected. For some employers, supporting workers with long-term conditions may be challenging due to staff shortages and financial uncertainties (12). Other employers may refuse to make the adjustments that employees with Long Covid would need to allow them to work (13). For governments, Long Covid has direct healthcare costs but also places a burden on support services, disability benefits, and labor participation and productivity of impacted individuals and their caregivers (14). Preliminary estimates suggest that the economic impact of Long Covid could reach between $864 billion to $1.04 trillion annually for OECD countries (15) with a global annual economic toll potentially around $1 trillion, equivalent to about 1% of the 2024 global GDP (2).

Third, healthcare systems, already strained by the pandemic, are further burdened by the ongoing medical care and specialist consultations required for Long Covid patients, leading to longer wait times, delays in essential care, and increased costs. The lack of standardized diagnostic and treatment protocols and pathways adds complexity for healthcare professionals (6). As a result, patients in the UK and many other countries often face unmet healthcare needs (16). Moreover, the increase in infection-associated non-communicable diseases (NCDs), such as diabetes, ‘obesity, hypertension, heart disease, diabetes, neurological disorders, or immune dysfunction’ resulting from SARS-CoV-2 infection further strains health systems by increasing the demand for long-term, chronic care and driving up healthcare costs (17).

Fourth, Long Covid is the first condition to emerge as patient-led/patient-named/patient-made. In this context, the idea of being patient made refers to the fact that it was named and conceptualized by patients. The initial mapping of Long Covid by Elisa Perego and Felicity Callard (18) underscored the importance of patient knowledge in conceptualizing and treating this condition in the early months and years of the pandemic, challenging traditional medical epistemic authority. Building on this foundation, numerous studies emphasize the importance of a patient-centered approach that values patient testimonies and addresses the holistic and diverse needs of those affected (19).

Fifth, qualitative social scientific studies have particularly explored the quality of life and the lived experiences of Long Covid patients, covering areas such as symptoms (20), rates of recovery and impact on daily activities (21), coping strategies, mental health and identity issues (10), healthcare experiences (16), stigma (22), online support groups (23), and work experiences (24). These studies support the biomedical and epidemiological research indicating decreased quality of life (25) and multifaceted long-term health effects following a SARS-CoV2 infection which varies significantly across individuals. Experiences with recovery reveal considerable barriers to accessing care, gaslighting and discrimination from healthcare professionals, extended periods of suffering, slow progress, and uncertainty about achieving full health. Seeking alternative care options, patients are turning to online resources, such as patients’ social media groups, which enable information exchange and peer support (26).

Sixth, and building on these challenges, studies also consistently highlight the substantial burden of psychosocial challenges, with over three-quarters of patients reporting a moderate to severe impact on their overall well-being (4). These challenges include changes in individuals’ sense of identity and self, capacity to work, carry out family roles, manage daily tasks and socialize. This, in turn, has repercussions for their families, caregivers, and communities (27). Many patients face social exclusion, isolation, and stigma, often from health care providers and encounter societal barriers to the inclusion of individuals with disabilities and chronic illnesses (10).

Finally, scholars working on Long Covid point to the ongoing challenges of a lack of reporting of, and data on, Long Covid cases and its impact, which influences and limits government’s capacity to respond (28).

To summarize, existing research points to patient experiences, public health impacts, a lack of standardized diagnostic and treatment protocols and pathways and the need for patient involvement as key areas of concern. What is not as clear is the extent to which these priorities are mirrored in the concerns expressed by those researching and living with Long Covid. Our agenda-setting exercise aims to address this gap. There are a few survey-based studies and systematic reviews highlighting the priorities that researchers should address in future research on Long Covid (20, 29), some emphasizing social scientific research in particular (30). However, most of these studies focus specifically either on patients’ perspectives or healthcare professionals’ perspectives. Al-Aly and his co-authors (2) in a recent Nature review laid out a research and policy roadmap for Long Covid, outlining both biomedical and social scientific priorities, but their framework was based on an assessment of evidence and policy gaps, alongside their clinical, research, and policy experience, developed in partnership with patients. Our research adopts a different approach by utilizing participatory research methods and centring the voices of a broad spectrum of individuals affected by Long Covid and involved in developing understandings of and responses to the illness, − as both participants and co-authors - including patients, families, carers, healthcare professionals, academics, private and public sector professionals, and volunteers from Long Covid charities and support groups. In this context, participatory research methods, well-established in human geography, anthropology, sociology and related disciplines, can play a role by involving participants in shaping research direction, including the selection of topics and questions.

## Methods

This study draws on two consecutive online surveys conducted in March and April 2024. The exercise comprised of four phases: survey design and participant recruitment, identifying social scientific research priorities, collation and prioritization of questions, and collaborative drafting of agenda.

### Stage 1: survey design and participant recruitment

The two surveys were conducted using purposive sampling, a technique to identify and select the most information-rich cases, including individuals or groups that have experience with a specific social phenomenon (31). Purposive sampling allowed us to focus on specific groups whose experiences and expertise are particularly relevant for Long Covid research priorities and ensured that our sample includes individuals who can provide insights into the various dimensions of the condition (32). Participant selection and recruitment was facilitated by the fact that the network of patients, organizations and professionals interested in Long Covid in the UK is relatively small and well-connected. Long Covid Support, a collaborator on this study, had established strong relationships within this community, and we were able to use this network, combined with the research team and advisory board’s network to recruit the identified participants. We identified a list of 48 people, seeking to ensure representation from across relevant interest groups, contacted them via email, and gave them the option to share this with their Long Covid contacts, which increased the final number of survey participants. For the survey, our inclusion criteria were that all participants will be over 18 and able to give informed consent, had an interest in Long Covid, either as a person with Long Covid or someone who works with people with Long Covid or conducts research into Long Covid and well enough to participate in planned activities (self-determined) on the day. We excluded participants who were under 18, unable to give informed consent. The number of participants is appropriate and justified given the nature and aims of our research. The primary objective was not to achieve broad generalisability, but rather to gain insights from different perspectives, targeting participants whose experiences, roles, or perspectives are deeply embedded within the community. Hence, the value of the data is not dependent on a large sample size, but on the relevance and depth of information provided by participants who are well-positioned to speak to Long Covid research priorities. It is also worth noting the numbers of participants here are like those seen in other similar exercises (33).

In total 57 participants contributed to our first online survey and 66 to our second survey (for details on participants’ roles in the community, illness severity, and demographic information, please refer to Supplementary Table 2). The participants included people with Long Covid (77.3%), carers (10.9%), close friends or family members (22.95%), colleagues or employers (6.0%), health professionals working with Long Covid patients (8.37%), academic researchers studying Long Covid (17.6%), and members of Long Covid support groups/charities (27%). Some participants identified with more than one of these categories, for example, it was common to find that professionals working in this space either currently live with Long Covid or have experienced it in the past. Additionally, we found that several people with Long Covid also had family members with the condition. Recognizing the challenges some patients face in obtaining a Long Covid diagnosis, we aimed to include individuals regardless of whether they had received a formal diagnosis. In the first survey, all Long Covid patients were formally diagnosed. In the second survey, 82.3% had a formal diagnosis, 15.7% did not, and 2.0% selected prefer not to say.

The severity of Long Covid varies widely, and the respective challenges and issues can vary considerably in line with this. In our sampling we aimed to include patients with a range of severities of illness. To assess whether we achieved this, participants with Long Covid responded to an additional question on their condition based on the Post-COVID Functional Status (PCFS) scale (34). We had representation from individuals across all severity levels, including those who improved and regained functional capacity, and those who had not. Feedback from our first survey revealed issues with the PCFS scale as participants felt that it did not adequately differentiate between varying severity levels of Long Covid and that the inclusion of mental health symptoms was problematic, potentially reinforcing the notion that Long Covid symptoms are merely psychological. In response, for the second survey, we revised the scale by removing the terms relating to mental health and clarified that our intentions were only to approximately capture the spectrum of Long Covid severity. The discussion also alerted us to a longer-term issue with respect to finding a balance between recognizing that Long Covid can have significant impacts on mental health, which requires appropriate recognition and support, and the risks any focus on mental health can risk playing into a psychosocial model of Long Covid which many patients have found potentially stigmatizing and dismissive of their symptoms.

Hossain et al.’s (1) review of the qualitative evidence on Long Covid suggested that underrepresented communities, such as people of color and gender minorities, may face additional health and social inequities that impact their experience with Long Covid. They argued that future research should therefore aim to include these groups to promote research equity. Smyths et al. (35) have since also highlighted the underrepresentation of these groups in Long Covid research. Responding to this, we intentionally included representation from ethnic minorities, drawing on our networks and collaborators. In total, 17.9% of participants in the first survey and 20.3% in the second survey identified as part of a minority ethnic group. We did not include a question on gender in the survey, although given evidence women are disproportionality affected by Long Covid, on reflection we think it would be valuable to add this variable to future research (36). Women’s experiences of Long Covid have formed the focus of our earlier work (19, 56).

Representation across regions of the UK was considered, including devolved nations. We also ensured representation across various age groups (see Supplementary Table 2). While a significant portion of participants were from older age groups (over 50% of participants were between 45 and 64 years, see Supplementary material), reflecting the demographics of professionals in different career fields, we also specifically sought out younger patients to include this perspective. Due to the small sample size, our study did not analyze correlation between participants’ demographics and their respective answers.

While our list may not encompass the entire spectrum of relevant stakeholders, it nevertheless offers sufficient diversity to fulfill our primary objective: broadening the scope and inclusivity of social scientific inquiries into Long Covid.

### Stage 2: identifying social scientific research priorities

**Figure 1:**
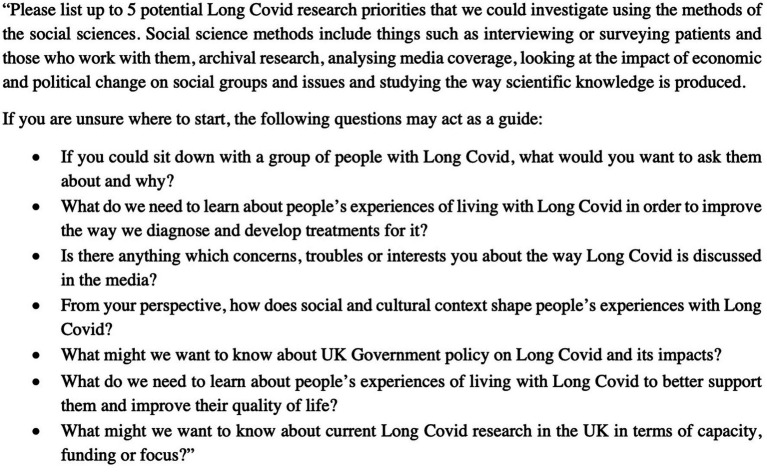
Survey 1 questions.

The first survey was created on the software JISC (Bristol, United Kingdom), provided by the University of Oxford. Three questions regarding socio-demographic information, relationship to Long Covid, and Long Covid health status were asked first (see Supplementary Tables). Participants were then requested to submit a list of (up to) five most important research questions regarding Long Covid. The sole restriction, aside from the focus on Long Covid, was that the queries should be conducive to social-scientific investigation. There were variations in the number of responses, and not all participants listed five questions. To ensure clarity about what social science research entails, we also provided a set of questions as prompts. An outline of the first survey is provided in Figure 1.

### Stage 3: collation and prioritization of questions and research topics

**Figure 2:**
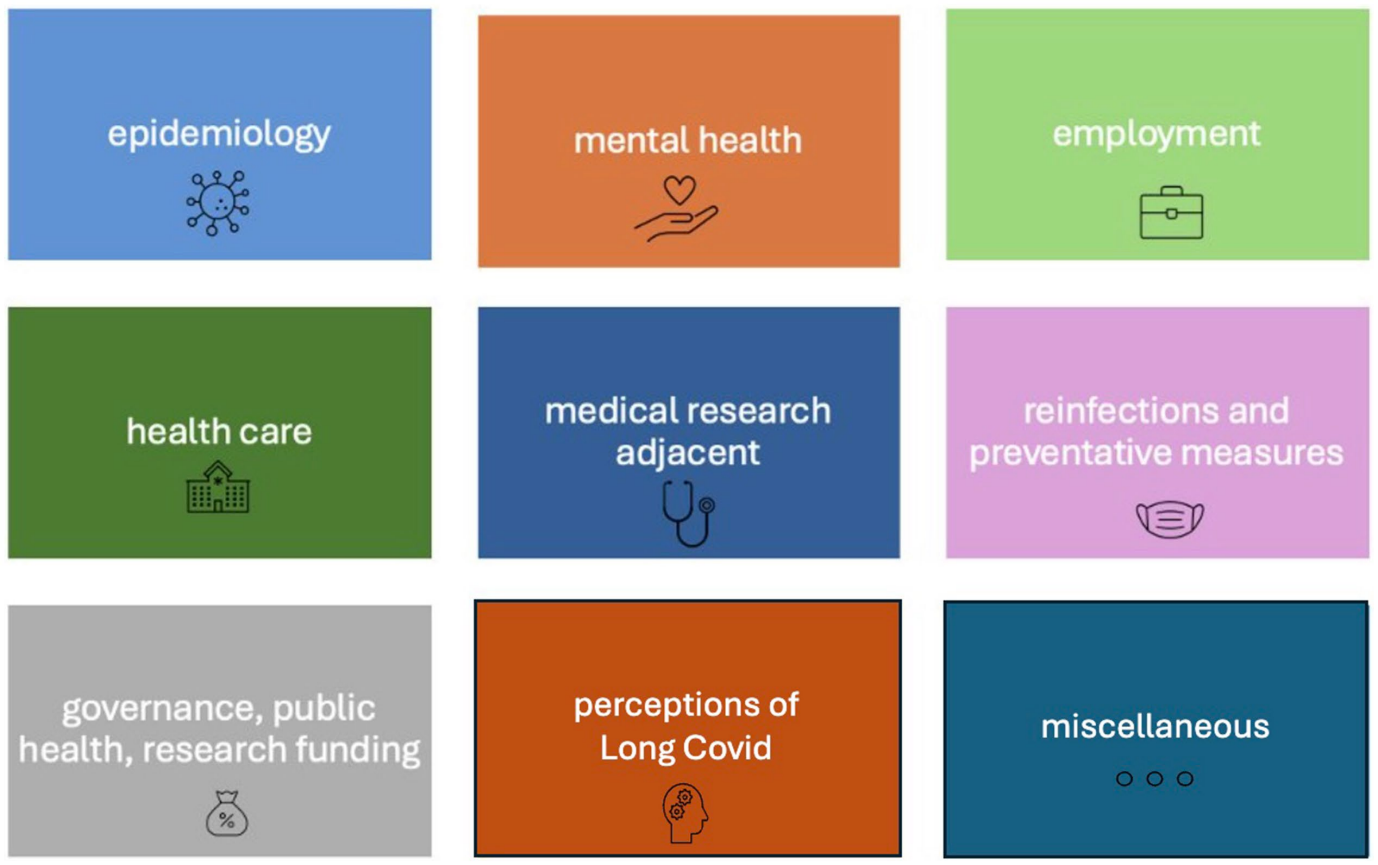
Nine research topics.

The research team collated and refined the submitted questions into a list of 55 questions. This entailed some editing, including bringing comparable questions together, removing questions that could not be answered by social science research and ensuring the questions were presented in a suitable format. An initial list was prepared by three of the co-authors (the two first authors and the last author) and distributed to a group of the co-authors for feedback, followed by adjustments. Discrepancies within the team were resolved through repeated discussion and reflection. We then grouped the 55 questions into the following nine research topics to aid responding to the second survey: epidemiology, medical research adjacent, reinfections and preventative measures, employment, healthcare, mental health, government response/public health/research funding, perception of Long Covid, miscellaneous (see Figure 2). While these topics are at first sight not social scientific research questions, nor ones which can be exclusively addressed through social science, we considered the ways that social sciences may help understand some aspects of these questions, including the use of social methods (interviews and surveys) and critical social science approaches to centring patients’ experiences in research.

In a second survey, the compiled list of the 55 potential research questions was distributed to participants mapped under the nine topics to better depict the broader contexts of the individual questions. The participants were asked to select 10 questions they found most important (for the full list of the questions and the number of ‘votes’ each received see Supplementary Table 3). For this survey, Microsoft Forms software was used. Again, questions regarding socio-demographic information, participant’s relationship to Long Covid, and their health status related to Long Covid were asked first. We then asked participants to choose up to 10 research questions that they deemed most important, stressing that while the questions were categorized into topics, there was no obligation to select a question from every topic and it was possible to choose several questions from one topic. Finally, we selected questions that received at least 23 votes regardless of topic, representing more than 50% of participants, resulting in a list of nine research questions. The topics were only used organize the material for the survey and were not included in the further discussion of the questions.

In a continuation of the collaborative approach we have adopted throughout this study, we also offered the contributors of the study the option to participate in the writing of this paper reporting our findings, and those who opted to do so are included in the list of authors above.

## Results

**Figure 3:**
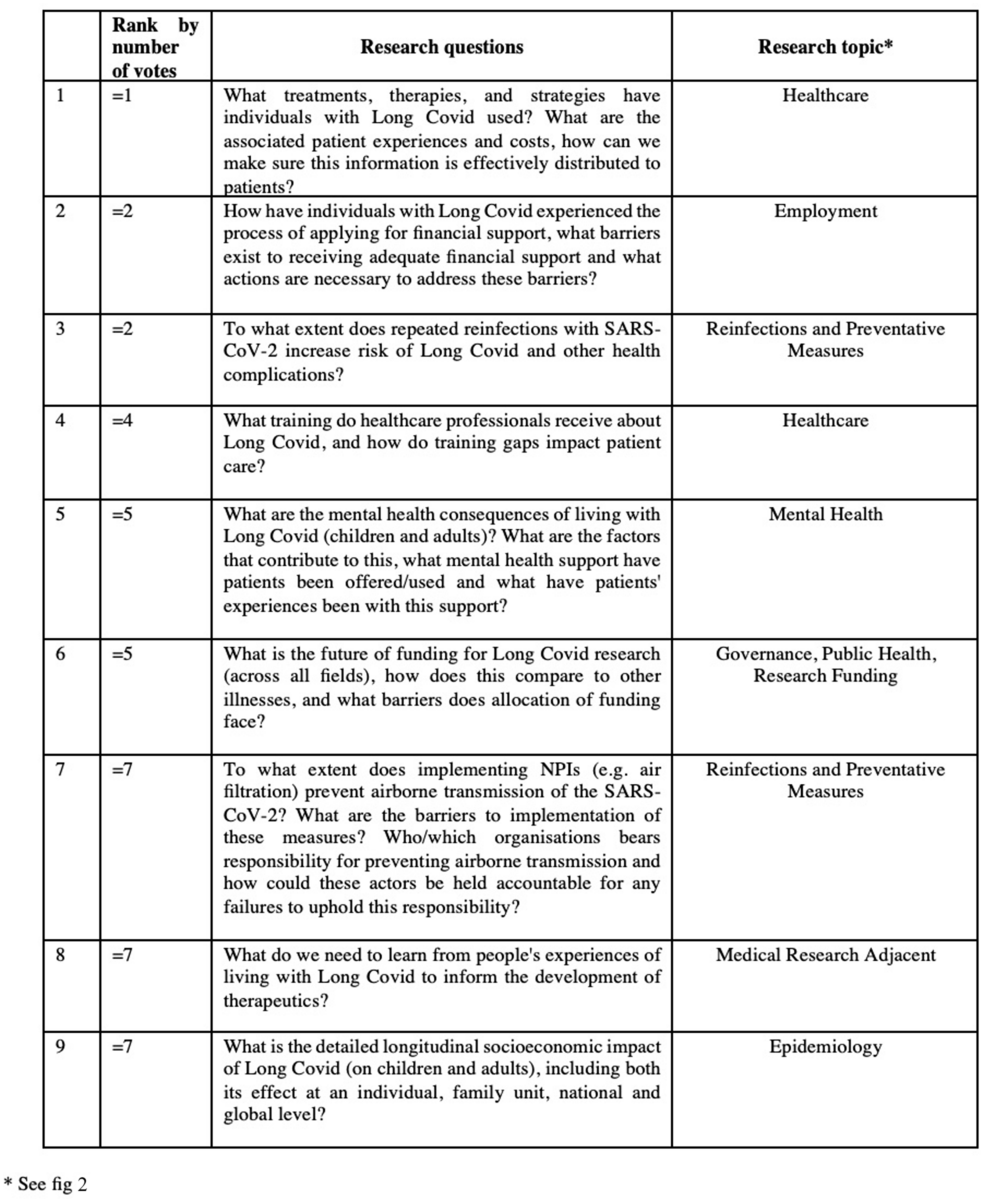
Nine highest-ranked research questions for social scientific research.

The results of our two-survey exercise were a ranked list of all the research questions (see Supplementary Table 3). The nine highest-ranked research questions are listed below (Figure 3).

## Discussion

In this section we discuss the nine highest-ranked research questions. We have grouped the questions according to themes in order to structure the discussion which follows. We have also included the ranking of the questions, by number of participant votes, in brackets alongside the question number, from 1 being the most important to 10 being the least important.

Questions 1(1), 8 (=7) and 4 (4):

- What treatments, therapies, and strategies have individuals with Long Covid used? What are the associated patient experiences and costs and how can we make sure this information is effectively distributed to patients?
- What do we need to learn from people’s experiences of living with Long Covid to inform the development of therapeutics?
- What training do healthcare professionals receive about Long Covid, and how do training gaps impact patient care?

Together, these questions address the need for a well-informed healthcare system where both patients and healthcare providers are equipped with the knowledge and resources to manage Long Covid effectively.

It is evident from the literature that care for individuals with Long Covid currently differs greatly across various settings and practitioners (4). COVID-19 has created significant challenges for clinicians in managing Long Covid, who must grapple with a lack of evidence to support Long Covid treatment, an absence of standardized care pathways and limited resources (see for example Greenhalgh et al. (37) for an update for primary care). This is further compounded by the extra strain placed on healthcare systems as a result of the pandemic. Additionally, findings from research into myalgic encephalomyeitis (ME), a chronic illness sharing some similarities with Long Covid in terms of symptoms, indicate that many health care practitioners feel their medical education did not sufficiently prepare them for diagnosing and managing chronic illnesses according to current guidelines (38). Social science has played a key role here in highlighting how the lack of widespread recognition and understanding of Long Covid can lead to a form of epistemic injustice, where clinicians fail to recognize and believe patients, leaving them feeling unheard, unsupported and gaslighted (28).

Understanding patient experiences and the effectiveness of various treatments and strategies can help ensure that successful approaches are recognized and shared widely (5). Social scientists can help by systematically collecting, analyzing and disseminating patients’ narratives about their individual treatment and revealing the variety of treatments used, including (in addition to medicines and supplements) machines such as pacing monitors and vagus nerve stimulators (39). Developing effective ways to disseminate this information and equip healthcare professionals to manage Long Covid is essential for ensuring that this knowledge reaches those who need it most (34). This can lead to more standardized care, better patient management, and ultimately, a reduction in the burden of Long Covid on both individuals and healthcare systems. This is particularly important with some Long Covid clinics already closing and patients being redirected to other services where staff have not had any training on Long Covid.

Question 2 (=2) and 9 (=7):

- How have individuals with Long Covid experienced the process of applying for financial support, what barriers exist to receiving adequate financial support and what actions are necessary to address these barriers?
- What is the detailed longitudinal socioeconomic impact of Long Covid (on children and adults), including both its effect at an individual, family unit, national and global level?

These two questions examine the significant socioeconomic impact of Long Covid on individuals and its broader systemic consequences, as well as the support available to those affected.

The economic impact of Long Covid is substantial and increasing, with one study finding 24.5% of individuals with Long Covid reducing their paid work hours, 28.2% leaving paid work altogether, and average monthly income falling by 24.5%. Additionally, 31.7% required informal care (27). These findings align with previous UK studies, which similarly reported significant work reductions, job losses, and economic strain among Long Covid patients (40). Long Covid might also have a financial impact, particularly on those who are not employed, as managing symptoms can incur additional costs (medical costs, paying for additional care or support) and potentially delay or prevent re-entering the job market (41). In addition to medical costs, paying for masks, nasal sprays and high-efficiency particulate air (HEPA) filters increases individual costs to mitigate against repeated infections (including treatment costs discussed in the previous section). The financial burden is likely to be unevenly distributed and to be exacerbated by existing health and economic inequalities. Furthermore, the changing political landscape can impact service provision.

Focusing on the UK, the recent change in administration, with the center-left Labor government coming into power in July 2024 has added additional uncertainty with respect to the social security landscape, and how this may impact the ability of people with Long Covid to access welfare support: conditionality and sanctions policies exist, and we do not know whether people with Long Covid currently or in the future will be expected to look for work when it is not safe for them to do so. There is limited knowledge regarding whether Long Covid is linked to increased reliance on welfare benefits, New Style Employment and Support Allowance, Universal Credit, and housing benefits, and what the experiences patients have had when applying for benefits. Recent reports have revealed the financial burden has led some patients to use loan sharks or to sell personal possessions (11). There is a key role here for social scientists in helping to understand patient experiences in applying for financial and employment support and identifying the barriers patients encounter, such as complex application processes, eligibility restrictions, or lack of awareness about available resources (11). For example, the Personal Independence Payment (PIP or ADP in Scotland) goes toward supporting the extra costs of health conditions or disability. It is not currently means-tested, but there are known barriers to claiming and it being awarded. Research is needed to inform policy changes and the development of more accessible and effective support systems which may reduce the financial strain on some individuals with Long Covid.

Regarding the broader systemic impact, studies suggest Long Covid has significantly impacted labor markets and economies in the UK (42) as well as globally. Long Covid is reducing people’s ability to work and therefore exacerbating labor shortages (2). In the UK, approximately 80,000 people have withdrawn from employment since the pandemic began due to Long Covid, representing 0.3% of the employed population (43). A 2022 study estimated the total cost in the US, including lost quality of life, earnings, and healthcare, could reach $3.7 trillion, equating to 17% of the 2019 GDP. This impact is comparable to the global 2008 Great Recession (2). For OECD countries, excluding healthcare costs, Long Covid may cost between $864 billion and $1.04 trillion annually due to decreased quality of life and labor participation (15). Globally, Long Covid’s annual economic toll could be around $1 trillion, or 1% of the 2024 global GDP (2).

Question 3 (=2):

- To what extent do repeated reinfections with SARS-CoV-2 increase risk of Long Covid and other health complications?

This question reflects concerns about repeated infections due to continued COVID-19 transmission and its potential effects. The risk that repeated infections pose is still not clear (44) but preliminary studies and patient experience suggest reinfection for those who have Long Covid can exacerbate the severity of the illness they are experiencing (45). Whether reinfections increase the risk of Long Covid is still contested, but the evidence is growing (37). Greenhalgh et al. (46) stress the importance for people with Long Covid not to get reinfected as it can prolong recovery. In a population of veterans, Bowe et al. (3) found that even 6 months after reinfection, there was an excess risk of outcomes such as heart disease, lung problems, diabetes, fatigue, and neurological disorders. However, recent data from the UK Office of National Statistics indicates that the risk of developing Long Covid decreases with subsequent infections (47). Adults had a 4% risk of developing Long Covid after a first infection, which declined to 2.4% after reinfection. For children and young people, the risk of Long Covid after a first infection was 1% and remained relatively unchanged after reinfection. This highlights the need for further investigation to fully understand the relationship between reinfections and Long Covid. Clarifying these risks is important for informing public health strategies and managing the long-term impacts of COVID-19 on the population. Social scientific research may help explore these questions through collaborative research with medical researchers, for example, combining narrative and longitudinal research to explore the relationship between how risks may be differentially perceived and evidenced by patients and epidemiologists.

Question 5 (5)

- What are the mental health consequences of living with Long Covid (children and adults)? What are the factors that contribute to this? What mental health support have patients been offered/used and what have patients’ experiences been with this support?

A number of studies have demonstrated the negative emotional impact of Long Covid with evidence indicating a decline in quality of life and mental health compared to those without. Figures indicate more than 87% of people with Long Covid have mental health symptoms (48). Prevalent pathologies include anxiety, depression, sleep disorders, PTSD, and mood fluctuations (49). These have also been observed in affected children (50). The precise causes of these mental health issues remain unclear, with possibilities including whether they are a psychological impact of the disease or if they involve physical changes in the brain. Patients report dissatisfaction with healthcare, anxiety about reinfection, and emotional distress linked to physical limitations and financial strain (1). There is also a sense of loss, and many felt that their self-identity was deeply affected by the illness. They experienced a shift in how they viewed themselves, needing to reconsider their roles within family and work (51). There is also a strong stigma associated with Long Covid, with patients feeling a sense of shame and blame and fearing discrimination from family, employers and the wider community (22). This leads many people, for example, to not disclose their issues with Long Covid in the workplace (11).

Limited studies have addressed social aspects such as stigma, discrimination, and social support, with findings suggesting that social isolation and stigma exacerbate mental health challenges (52). Long Covid significantly impacts both individual lives and societal well-being. Understanding its effects on social interactions, such as lost friendships, strained relationships, and reduced networking ability, is crucial. Insight into the social responses, stigma, and the interplay between social consequences and health outcomes will aid in developing supportive interventions. Additionally, assessing the burden on caregivers, families, and social groups is essential.

Al-Jabr et al. (48) carried out a scoping review of interventions to support mental health in people with Long Covid, which highlighted a wide range of approaches. Some studies explored specific pharmacological products or dietary changes, but most utilized integrated treatments delivered by multidisciplinary teams, requiring active patient participation. All the interventions reviewed showed positive outcomes but the findings should be interpreted cautiously due to the limited quality and scale of most studies. More large-scale, high-quality trials are needed to identify effective strategies, particularly those addressing the varied symptoms of Long Covid. Social science could complement and extend this research by offering insights into contributing factors, the mental health support offered, and patients’ experiences with this support that are essential for addressing these issues effectively and improving overall care and outcomes for Long Covid patients (52).

Question 6 (=5):

- What is the future of funding for Long Covid research (across all fields), how does this compare to other illnesses, and what barriers does allocation of funding face?

As of 2020, the UK government has invested over £50 million into research focused on understanding Long Covid, its symptoms, and potential treatment, the vast majority of this being allocated in 2021. This funding has been channeled primarily through the National Institute for Health and Care Research and UK Research and Innovation (53), leading to the largest clinical trials on the subject and deeper understanding into the underlying biological mechanisms of the disease. From this work, it is evident that developing effective treatment may be possible. However, different symptom clusters and physiological markers will likely require tailored treatments, meaning there will not be a universal cure for Long Covid. This underscores the need for numerous well-designed trials, each focused on specific patient subgroups (2). Despite this progress, there is a growing concern over the long-term sustainability of this funding, especially as economic pressures and shifting priorities may divert resources elsewhere. Long Covid clinics will be closed or merged into other clinics, e.g., general rehab and applications for research funding have to compete with bids for other conditions. There are multiple risks to this happening, and the scale of the problem justifies continuing with the ring-fenced model. Researchers are calling for continued ring-fenced financial support for Long Covid research to inform targeted strategies to support recovery and mitigate wider socioeconomic impact.

Social science research could offer an understanding how financial commitment to Long Covid research compares with funding for other diseases, as well as identifying the barriers this research faces, which is important for a number of reasons. First, it allows us to gage whether Long Covid is receiving the appropriate level of attention and resources relative to its impact on public health. By comparing funding levels, we can assess whether Long Covid is being prioritized adequately in comparison to more established illnesses like cancer, heart disease, or diabetes, which have long benefited from substantial research investments. Second, identifying barriers to Long Covid research funding, such as limited awareness, or the complex and varied nature of Long Covid symptoms, enables us to address these challenges strategically. By understanding these dynamics, we can better advocate for the level of funding that Long Covid research deserves.

Question 7 (=7):

- To what extent does implementing Non-Pharmaceutical Interventions (e.g., air filtration) prevent airborne transmission of the SARS-CoV-2? What are the barriers to implementation of these measures? Who/which organizations bears responsibility for preventing airborne transmission and how could these actors be held accountable for any failures to uphold this responsibility?

The push for better indoor air quality stems from the understanding that SARS-CoV2, along with many other infectious diseases, is airborne and spreads primarily in indoor environments. Many people with Long Covid are fully aware that anyone is vulnerable and therefore they are desperate to avoid other people being affected as they are. Research has demonstrated that improving ventilation can significantly reduce infection risks; for instance, a study in Italian schools found that mechanical ventilation systems could lower students’ infection risk by up to 80% (54). Enhanced ventilation also decreases exposure to pollutants, such as fine particulates from wildfire smoke and cooking, volatile organic compounds leached from furniture, and allergy-causing molds and pollens (55). However, implementing effective air filtration systems presents substantial challenges, particularly in terms of cost. Retrofitting existing buildings with the necessary technology to achieve adequate clean air levels is projected to be expensive. Nonetheless, experts argue that the long-term benefits would far outweigh these costs (55). For example, pandemic and seasonal influenza outbreaks cost the United Kingdom an estimated £23 billion (US$27 billion) annually, but improved ventilation could potentially save the country £174 billion over 60 years (57). Despite these clear advantages, the responsibility for indoor air quality is often split across various government departments and professional bodies, making it implementation and enforcement challenging (55). Social science could offer insights into the institutional, economic, regulatory and socio-cultural barriers to improving indoor air quality.

## Conclusion

This paper aimed to identify key research questions for social scientific research that would reflect the needs and demands of the Long Covid community. Our study includes of course also some limitations. We are aware we cannot speak for everyone but have tried to gather insights from patients and other stakeholders, many of whom have conducted research in different areas on Long Covid for many years.

The study identified nine top research questions, which concerned (i) treatments, therapies, and strategies; (ii) financial support; (iii) repeated reinfections; (iv) training of healthcare professionals; (v) mental health impact; (vi) future of research funding; (vii) the airborne transmissions of COVID-19; (viii) developing therapeutics informed by patients’ experiences; and (ix) the socioeconomic impact of Long Covid.

Based on these questions, we draw our key messages from the agenda-setting exercise.

First, the agenda-setting exercise flagged the key role social science can play in future research on Long Covid. Social science offers valuable tools for understanding the broader societal and structural implications of the condition, including its impact on healthcare systems, employment policies, and social support mechanisms, highlighting the role of individual (and diverse) experiences. By broadening the research agenda to include social dimensions, future studies can offer a more comprehensive understanding of the condition and its far-reaching effects. Notably, some of the issues our survey contributors flagged regarding the risks of re-infection and the question of responsibility for indoor air quality did not emerge as key focuses in previous reviews (see literature review), highlighting the new perspectives and value offered by this collaborative approach. This is particularly important considering that recent research has revealed the harms to recovery if reinfected with COVID-19 (46).

Second, our survey demonstrated there is an urgent need to include the voices of a diverse range of individuals affected by Long Covid and those working in the Long Covid space. A key distinction of our exercise was the collaboration with Long Covid Support, with one of their members (and first author of this paper) joining our university research team, and the co-authorship of survey participants. Through Long Covid Support’s network and using purposive sampling, we targeted a range of both academic and non-academic groups with relevant expertise, in contrast with studies which tend to focus on specific medical or disciplinary fields or on specific user groups, e.g., patients or carers, or systematic reviews which only focus on academic research and thereby can exclude civil society groups, patient and carer perspectives Our sample included people with Long Covid, carers, family members, healthcare professionals, academic researchers and civil society group members. By carrying the exercise out in this way, we hope to improve the scope and inclusivity of social scientific inquiries into Long Covid. This is reflected in the breadth of issues raised in the survey, which range from questions focused on specific clinical interests (such as oral health), to questions about the economy to patient and carer experiences and concerns.

Many of the issues highlighted in our key questions echo those we outlined in our opening literature review, including gaps in biomedical research and the need for research funding, persistent challenges related to access to appropriate healthcare, gaps in medical education about the condition, the long-term impact on patients’ quality of life, and the impact on health systems and the economy. The consistency between our initial review and the feedback from participants underscores the need for urgent and sustained action in these areas. Policy makers could make use of this priority list to inform current and future priorities for funding and intervention. For healthcare organizations, the list acts as a useful guide as to where it is important to engage with social science research in the development of strategies and services for Long Covid patients. For community organizations, this research offers a useful model of collaborative agenda-setting which may be relevant for other health conditions.

## Data Availability

The original contributions presented in the study are included in the article/Supplementary material, further inquiries can be directed to the corresponding author.
