# Correction: Co-creating a social science research agenda for Long Covid

**DOI:** 10.3389/fpubh.2026.1748942

**Published:** 2026-07-01

**Authors:** Oonagh Cousins, Maaret Jokela-Pansini, Nisreen A. Alwan, Ella Barnard, Jenny Ceolta-Smith, Jo Dainow, Caroline Dalton, Gail Davies, Mark A. Faghy, Eileen Gilmour, Ian Patel, Ondine Sherwood, Lotus Westerhof, Beth Greenhough

**Affiliations:** 1 Long Covid Support, London, United Kingdom; 2 School of Geography and the Environment, University of Oxford, Oxford, United Kingdom; 3 Department of Geography, University of Zurich, Zürich, Switzerland; 4 School of Primary Care, Population Sciences and Medical Education, Faculty of Medicine, University of Southampton, Southampton, United Kingdom; 5 University Hospital Southampton NHS Foundation Trust, Southampton, United Kingdom; 6 Patient Author, London, United Kingdom; 7 College of Health Wellbeing and Life Sciences, Sheffield Hallam University, Sheffield, United Kingdom; 8 Geography, Faculty of Environment, Science and Economy, University of Exeter, Exeter, United Kingdom; 9 Human Science Research Centre, University of Derby, Derby, United Kingdom; 10 Patient Author, Kilmarnock, United Kingdom; 11 Patient Author, North Berwick, United Kingdom; 12 Long Covid SOS, London, United Kingdom; 13 Patient Author, Harderwijk, Netherlands

**Keywords:** Long Covid, COVID-19, social science, agenda-setting, survey, PPI, stakeholder engagement, co-creation

Authors Eileen Gilmour, Ian Patel and Lotus Westerhof were erroneously assigned to “Patient Author, London, United Kingdom”

Affiliation “Patient Author, Kilmarnock, United Kingdom” was omitted from the paper. This affiliation has now been added for author Eileen Gilmour.

Affiliation “Patient Author, North Berwick, United Kingdom” was omitted from the paper. This affiliation has now been added for author Ian Patel.

Affiliation “Patient Author, Harderwijk, Netherlands” was omitted from the paper. This affiliation has now been added for author Lotus Westerhof.

Affiliation “Long Covid Support” was omitted for author Jenny Ceolta-Smith. This affiliation has now been added for author Jenny Ceolta-Smith.

Author “Jenny Ceolta-Smith” was omitted as an author in the published article. The correct author list reads:

Oonagh Cousins^1^, Maaret Jokela-Pansini^2, 3*^, Nisreen A. Alwan^4, 5^, Ella Barnard^6^, Jenny Ceolta-Smith^1^, Jo Dainow^1^, Caroline Dalton^7^, Gail Davies^8^, Mark A. Faghy^9^, Eileen Gilmour^10^, Ian Patel^11^, Ondine Sherwood^12^, Lotus Westerhof^13^ and Beth Greenhough^2^

The Author Contributions Statement has been corrected to read:

OC: Formal analysis, Data curation, Writing – original draft, Writing – review & editing, Validation, Investigation, Methodology, Conceptualization, Funding acquisition, Project administration. MJ-P: Project administration, Writing – original draft, Data curation, Formal analysis, Methodology, Validation, Investigation, Supervision, Writing – review & editing, Conceptualization, Funding acquisition. NA: Conceptualization, Writing – original draft, Writing – review & editing, Validation. EB: Validation, Writing – review & editing. JC-S: Writing – review & editing. JD: Validation, Writing – review & editing. CD: Writing – review & editing, Validation. GD: Validation, Writing – review & editing. MF: Validation, Writing – review & editing. EG: Writing – review & editing. IP: Writing – review & editing. OS: Validation, Writing – review & editing. LW: Writing – review & editing, Validation. BG: Writing – original draft, Formal analysis, Conceptualization, Investigation, Project administration, Supervision, Writing – review & editing, Validation, Funding acquisition, Methodology, Data curation.

The original version of this article has been updated.

