# Supplementary material for: Co-creating a social science research agenda for Long Covid
Source: Front Public Health. 2025 Nov 6;13:1654488. doi: 10.3389/fpubh.2025.1654488 (PMC12631371; doi:10.3389/fpubh.2025.1654488)
Supplement: Supplementary file 1 [file Table_1.DOCX]

**Supplementary table 1 Demographic information of survey participants**

|  | **Survey 1** | **Survey 2** |
| --- | --- | --- |
|  | N=56 | N=64 |
| **Characteristic** | **N=%** | **N=%** |
| **Age** |  |  |
| 18-24 | 3 (5.4) | 3 (4.7) |
| 25-34 | 13 (23.2) | 10 (15.6) |
| 35-44 | 7 (12.5) | 14 (21.9) |
| 45-54 | 16 (28.6) | 15 (23.4) |
| 55-64 | 15 (26.8) | 19 (29.7) |
| 65-74 | 2 (3.6) | 3 (4.7) |
|  |  |  |
| **Location** |  |  |
| North-East & Cumbria | 1 (1.8) | 1 (1.56 (1) |
| North-West | 8 (14.3) | 18.75 (12) |
| Yorkshire & the Humber | 4 (7.1) | 10.94 (7) |
| East Midlands | 5 (8.9) | 9.38 (6) |
| West Midlands | 3 (5.4) | 4.69 (3) |
| London and South East | 22 (39.3) | 35.94 (23) |
| South West | 6 (10.7) | 9.38 (6) |
| Wales | 1 (1.8) | 1.56 (1) |
| Scotland | 4 (7.1) | 4.69 (3) |
| Northern Ireland | 1 (1.8) | 3.13 (2) |
| Other | 1 (1.8) | 0.00 (0) |
|  |  |  |
| **Ethnicity** |  |  |
| **White** | **46 (82.1)** | **51 (79.7)** |
| White British | 38 (67.8) | 44 (68.8) |
| White Irish | 1 (1.8) | 2 (3.1) |
| White Other | 7 (12.5) | 5 (7.8) |
| **Asian or Asian British** | **2 (3.8)** | **4 (6.3)** |
| Bangladeshi | 1 (1.8) | 1 (1.6) |
| Pakistani | 0 | 1 (1.6) |
| Indian | 1 (1.8) | 2 (3.1) |
| Chinese | 0 | 0 |
| Any other Asian background | 1 (1.8) | 1 (1.6) |
| **Black, Black British Caribbean, African** | **1 (1.8)** | **0** |
| Black African | 0 | 0 |
| Black Carribean | 1 (1.8) | 0 |
| Other Black, Black British or Caribbean background | 0 | 0 |
| **Mixed or multiple ethnic groups** | **6 (10.7)** | **7 (10.9)** |
| Mixed White and Black African | 0 | 2 (3.1) |
| Mixed White and Black Caribeean | 1 (1.8) | 1 (1.6) |
| Mixed White and Asian | 3 (5.4) | 2 (3.1) |
| Mixed Other | 1 (1.8) | 2 (3.1) |
| **Other** | 1 (1.8) | **1 (1.6)** |
| Prefer not to say | 0 | 1 (1.6) |
| Other | 1 (1.8) | 0 |
