# Supplementary material for: Co-creating a social science research agenda for Long Covid
Source: Front Public Health. 2025 Nov 6;13:1654488. doi: 10.3389/fpubh.2025.1654488 (PMC12631371; doi:10.3389/fpubh.2025.1654488)
Supplement: Supplementary file 2 [file Table_2.DOCX]

**Supplementary table 2: Relationship to Long Covid, Long Covid diagnosis and severity of illness**

|  | **Survey 1** | **Survey 2** | |
| --- | --- | --- | --- |
|  | **N = 56** | **N=64** | |
|  | **N=(%)** | **N=(%)** | |
| **Relationship to Long Covid (multiple answers permitted)** |  |  | |
| A close friend or family member has Long Covid | 16 (28.6) | 11 (17.2) | |
| I am a carer for someone with Long Covid | 7 (12.5) | 6 (9.4) | |
| I am a colleague of/employ someone with Long Covid | 5 (8.9) | 2 (3.1) | |
| I am a medical professional working with Long Covid patients | 5 (8.9) | 5 (7.8) | |
| I am an academic researcher who studies Long Covid | 11 (19.6) | 10 (15.6) | |
| I have Long Covid (self-identified or formal diagnosis) | 42 (75) | 51 (79.7) | |
| I work at a NHS Long Covid Clinic | 1 (1.8) | 2 (3.1) | |
| I work for a private sector organisation related to Long Covid | 1 (1.8) | 2 (3.1) | |
| I work/volunteer for a Long Covid charity or support group | 20 (35.7) | 12 (18.7) | |
| Other | 4 (7.1) | 1 (1.6) | |
|  |  |  |  |
|  |  |  |  |
|  |  |  |  |
| **Number with Long Covid** | 42 (75) | 51 (79.7) |  |
|  |  |  |  |
| **Formal Diagnosis** |  |  |  |
| Yes | 42 (75) | 42 (82.3) |  |
| No | 0 | 8 (15.7) |  |
| Prefer not to say | 0 | 1 (2) |  |
|  |  |  |  |
| **Severity of Illness** |  |  |  |
| **Current Severity** |  |  |  |
| Level 0: I have no limitations in my everyday life and related to the infection. | 0 | 0 |  |
| Level 1: I have negligible limitations in my everyday life as I can perform all usual duties/activities, although I still have persistent symptoms. | 3 | 4 |  |
| Level 2: I suffer from limitations in my everyday life as I occasionally need to avoid or reduce usual duties/activities or need to spread these over time due to symptoms. I am, however, able to perform all activities without any assistance. | 5 | 6 |  |
| Level 3: I suffer from limitations in my everyday life as I am not able to perform all usual duties/activities due to symptoms. I am, however, able to take care of myself without any assistance. | 24 | 27 |  |
| Level 4: I suffer from severe limitations in my everyday life: I am not able to take care of myself and therefore I am dependent on nursing care and/or assistance from another person due to symptoms. | 10 | 14 |  |
|  |  |  |  |
| **At Maximum Severity (potentially the same as current severity)** |  |  |  |
| Level 0: I have no limitations in my everyday life and no symptoms pain, depression or anxiety related to the infection. | 0 | 0 |  |
| I have negligible limitations in my everyday life as I can perform all usual duties/activities, although I still have persistent symptoms. | 2 | 2 |  |
| I suffer from limitations in my everyday life as I occasionally need to avoid or reduce usual duties/activities or need to spread these over time due to symptoms. I am, however, able to perform all activities without any assistance. | 2 | 3 |  |
| I suffer from limitations in my everyday life as I am not able to perform all usual duties/activities due to symptoms. I am, however, able to take care of myself without any assistance. | 10 | 16 |  |
| I suffer from severe limitations in my everyday life: I am not able to take care of myself and therefore I am dependent on nursing care and/or assistance from another person due to symptoms. | 28 | 30 |  |
