# Supplementary material for: Co-creating a social science research agenda for Long Covid
Source: Front Public Health. 2025 Nov 6;13:1654488. doi: 10.3389/fpubh.2025.1654488 (PMC12631371; doi:10.3389/fpubh.2025.1654488)
Supplement: Supplementary file 3 [file Table_3.DOCX]

Supplementary table 3: Survey 2 Full list of research priorities

| **Priority number** | **Rank (by number of votes)** | **Survey question number** | **Research questions** | **Topic** | **Number of participants** |
| --- | --- | --- | --- | --- | --- |
| 1 | 1 | 13.1 | What treatments, therapies, and strategies have individuals with Long Covid used? What are the associated patient experiences and costs, how can we make sure this information is effectively distributed to patients? | Healthcare | 32 |
| 2 | 2 | 12.3 | How have individuals with Long Covid experienced the process of applying for financial support, what barriers exist to receiving adequate financial support and what actions are necessary to address these barriers? | Employment | 30 |
| 3 | =2 | 11.1 | To what extent does repeated reinfections with SARS-CoV-2 increase risk of Long Covid and other health complications? | Reinfections and Preventative Measures | 30 |
| 4 | 4 | 13.2 | What training do healthcare professionals receive about Long Covid, and how do training gaps impact patient care? | Healthcare | 27 |
| 5 | 5 | 14 | What are the mental health consequences of living with Long Covid (children and adults)? What are the factors that contribute to this, what mental health support have patients been offered/used and what have patients' experiences been with this support? | Mental Health | 24 |
| 6 | =5 | 15.5 | What is the future of funding for Long Covid research (across all fields), how does this compare to other illnesses, and what barriers does allocation of funding face? | Governance, Public Health, Research Funding | 24 |
| 7 | 7 | 11.4 | To what extent does implementing NPIs (e.g. air filtration) prevent airborne transmission of the SARS-CoV-2? What are the barriers to implementation of these measures? Who/which organisations bears responsibility for preventing airborne transmission and how could these actors be held accountable for any failures to uphold this responsibility? | Reinfections and Preventative Measures | 23 |
| 8 | =7 | 10.1 | What do we need to learn from people's experiences of living with Long Covid to inform the development of therapeutics? | Medical Research Adjacent | 23 |
| 9 | =7 | 9.1 | What is the detailed longitudinal socioeconomic impact of Long Covid (on children and adults), including both its effect at an individual, family unit, national and global level? | Epidemiology | 23 |
| 10 | 10 | 12.2 | What does best practice look like when employers provide accommodations for employees with Long Covid, in terms of reasonable adjustments? | Employment | 21 |
| 11 | 10 | 17.7 | Why are severe forms of Long Covid neglected and inadequately addressed by policymakers, healthcare providers, and researchers. What measures are required to address this issue? | Miscellaneous | 21 |
| 12 | 12 | 15.1 | A comprehensive evaluation of the UK and devolved government's past and current response to Long Covid, across all government departments, in comparison to other diseases, and examination of any factors that contributed to the response. | Governance, Public Health and Research Funding | 19 |
| 13 | =12 | 16.2 | How does the public perceive Covid-19 and Long Covid, in comparison to other illnesses, what factors have contributed to this, and how can we alleviate a lack of awareness/any misunderstandings? | Perception of Long Covid | 19 |
| 14 | 14 | 13.9 | How can personalised treatment plans be better developed for Long Covid patients? | Healthcare | 18 |
| 15 | =14 | 13.11 | How well is clinical and qualitive research into Long Covid being translated into healthcare practice? | Healthcare | 18 |
| 16 | =14 | 15.5 | What is the future of funding for Long Covid healthcare services and what barriers does allocation of funding face? | Governance, Public Health and Research Funding | 18 |
| 17 | 17 | 13.3 | How accessible is the provision of healthcare and community support for Long Covid across the UK, what factors contribute to any variability and what strategies can be implemented to alleviate this? | Healthcare | 17 |
| 18 | =17 | 17.1 | What are the implications of the lack of a clear definition of, diagnostic test for or biomarkers for, Long Covid? | Miscellaneous | 17 |
| 19 | =17 | 13.14 | Why do some healthcare professionals appear to think patients are unreliable witnesses for their own health? | Healthcare | 17 |
| 20 | 20 | 15.3 | How can policymakers be encouraged to adopt a more holistic approach to policy evaluation in regards to Long Covid, considering long-term socioeconomic benefits and costs? | Governance, Public Health and Research Funding | 16 |
| 21 | =20 | 17.13 | How can we ensure that patient voices are centred in Long Covid research? | Miscellaneous | 16 |
| 22 | =20 | 11.3 | How does the ongoing risk of reinfection affect those with Long Covid? | Reinfections and Preventative Measures | 16 |
| 23 | =20 | 16.1 | How is Long Covid being represented in the media, what are the impacts of this on patients and publics and what, if any changes might be needed? | Perception of Long Covid | 16 |
| 24 | =20 | 3 | What population level data on who is affected by Long Covid has been/is currently being collected by the UK government? | Epidemiology | 16 |
| 25 | 25 | 13.4 | What are Long Covid patient experiences of seeking care, treatment and support through the NHS and the private sector, and how does this experience affect future healthcare-seeking behaviour? | Healthcare | 15 |
| 26 | =25 | 13.13 | What are the attitudes of health professionals towards Long Covid patients, what are the factors contributing to this, and how might we alleviate any stigma? | Healthcare | 15 |
| 27 | 27 | 17.8 | Investigation into the progression of the disease including range of symptoms, fluctuations of disease, functional impairment and range of recovery. | Epidemiology | 14 |
| 28 | =27 | 15.6 | What strategies can patients, patient groups, and other advocates for Long Covid use to enhance the UK government, devolved government and industry's approach to addressing Long Covid, identifying key stakeholders or organisations, and determining effective leverage points for advocacy efforts? | Governance, Public Health and Research Funding | 14 |
| 29 | =27 | 10.2 | Why historically has there been limited medical research on post-acute infection syndromes? | Medical Research Adjacent | 14 |
| 30 | 30 | 17.2 | How could we improve the language associated with chronic illnesses, including conditions' name, to capture and communicate the complexity of these conditions? | Miscellaneous | 12 |
| 31 | =30 | 13.8 | To what extent can international knowledge-sharing initiatives improve the management and treatment approaches for post-acute-infection-syndromes? | Healthcare | 12 |
| 32 | =30 | 13.16 | What factors contribute to the lack of recognition of Long Covid in certain patients by both the patients themselves and by healthcare professionals. What measures are needed to address this? | Healthcare | 12 |
| 33 | 33 | 13.7 | What are the experiences of individuals with Long Covid from ethnic minority groups and how should this inform healthcare provision? | Healthcare | 11 |
| 34 | =33 | 17.6 | What factors contribute to the chronic nature of Long Covid? | Miscellaneous | 11 |
| 35 | 35 | 13.5 | How can we better assess the effectiveness of Long Covid clinics? | Healthcare | 10 |
| 36 | =35 | 16.3 | How do families, friends, partners and colleagues of Long Covid patients perceive Long Covid and is there sufficient provision of resources to educate and support these groups? | Perception of Long Covid | 10 |
| 37 | =35 | 13.12 | How would individuals living with Long Covid like to see their needs addressed? | Healthcare | 10 |
| 38 | =35 | 17.4 | What is the social impact on individuals (children and adults) living with Long Covid and their family units, over the time course of the illness, how does this affect Long Covid patients, and what support can be provided to alleviate negative impacts? | Miscellaneous | 10 |
| 39 | 39 | 17.12 | How can cross- and mutli- disciplinary collaboration improve research into Long Covid and support for those affected? | Miscellaneous | 9 |
| 40 | =39 | 15.4 | What factors contribute to the collective memory of the pandemic and why/how does collective amnesia arise, and with that in mind, what can be done to stop Long Covid being forgotten about by policy makers/researchers/healthcare professionals? | Governance, Public Health and Research Funding | 9 |
| 41 | 41 | 13.6 | How can patient consultations be integrated to ensure a more effective and tailored healthcare delivery? | Healthcare | 8 |
| 42 | =41 | 15.2 | How has the experience of Long Covid influenced perceptions and attitudes towards other post-acute infection syndromes, particularly among policymakers and researchers? | Governance, Public Health and Research Funding | 8 |
| 43 | =41 | 11.2 | What factors influence an individual's adoption of preventative measures against SARS-CoV-2 reinfections? How do these behaviours differ among those with and without Long Covid? What strategies can be used to alleviate any challenges faced by individuals who choose to use precautions? | Reinfections and Preventative Measures | 8 |
| 44 | =41 | 17.10 | What insights can be gained from comparative studies between Long Covid and other post-acute-infection syndromes and chronic illnesses (such as ME/ CFS), and how can these inform strategies for research? | Miscellaneous | 8 |
| 45 | =41 | 15.7 | What strategies have other patient advocacy groups used to successfully achieve development of treatments for other illnesses? | Governance, Public Health and Research Funding | 8 |
| 46 | 46 | 17.11 | How do fluctuations in the severity of Long Covid impact patients' quality of life, and how can this understanding inform healthcare and support provision? | Healthcare | 7 |
| 47 | =46 | 12.1 | What are employers' attitudes to Long Covid, across a range of sectors? | Employment | 7 |
| 48 | =46 | 9.2 | What is the socioeconomic profile of those with Long Covid and what factors contribute to this? | Epidemiology | 7 |
| 49 | 49 | 17.9 | How can we make the findings and data from Long Covid research more accessible? | Miscellaneous | 5 |
| 50 | =49 | 13.15 | What insights can we learn from experience of Long Covid about the reception of healthcare professionals to a new or unexplained health problem? | Healthcare | 5 |
| 51 | =49 | 17.5 | What role do community networks play in the lives of Long Covid patients, including patient-peer relationships. How do these networks contribute to information access, mental health and overall well-being? | Miscellaneous | 5 |
| 52 | 52 | 13.5 | How can we better assess the effectiveness of Long Covid clinics? | Healthcare | 4 |
| 53 | =52 | 17.3 | How does gender shape prevalence, experience and perception of Long Covid and other similar conditions (e.g. ME/CFS)? | Miscellaneous | 4 |
| 54 | =52 | 13.10 | What are the current oral health statuses and hygiene practices among individuals, in relation to the severity and prediction of Long Covid. What are the barriers to improving oral health? | Healthcare | 4 |
| 55 | 55 | 16.4 | How do Long Covid patients perceive Long Covid? | Perception of Long Covid | 3 |
